# Management of Chronic Hypoparathyroidism and Hyperparathyroidism in Pregnancy/Lactation

**DOI:** 10.1007/s11914-026-00979-w

**Published:** 2026-08-01

**Authors:** Stefan Pilz, Daniel Arian Kraus, Lisa Schmitt, Miriam Meister, Christina M. Berr, Maria P. Yavropoulou, Uwe Riedmann

**Affiliations:** 1 Department of Internal Medicine, Division of Endocrinology and Diabetology, Medical University of Graz, Auenbruggerplatz 15, Graz, 8036 Austria; 2 Department of Endocrinology, I. Medical Clinic, University Hospital Augsburg, Augsburg, Germany; 3 Endocrinology Unit, First Department of Propaedeutic and Internal Medicine, Medical School, National and Kapodistrian University of Athens, LAIKO University Hospital, Athens, Greece

**Keywords:** Parathyroid hormone, PHPT, Hypoparathyroidism, Calcium, Vitamin D, Hyperparathyroidism, Pregnancy

## Abstract

**Purpose of Review:**

We summarize and discuss evidence on management of chronic hypoparathyroidism (HypoPT) and primary hyperparathyroidism (PHPT) in pregnancy and lactation to provide guidance for clinical care.

**Recent Findings:**

Women with HypoPT are at increased risk of certain pregnancy complications, though most pregnancies are uncomplicated. Conventional therapy with activated vitamin D and calcium is continued throughout pregnancy, but there are unpredictable changes in the required dosages warranting regular surveillance during pregnancy (e.g., all 3 to 4 weeks). Data on parathyroid hormone (PTH) replacement therapy in pregnancy are limited, but a few case reports suggest favourable outcomes with this treatment. During lactation, dosage requirements for conventional therapy of HypoPT are often reduced and usually normalize again after weaning. In women with PHPT, surgical treatment should be pursued before conception. PHPT is not associated with adverse pregnancy outcomes in women with mild hypercalcemia but maternal and fetal complications significantly increase with higher calcium concentrations. There is no clear threshold for this risk increase, but several studies and expert groups support a cut-off concentration of about 2.85 mmol/L in albumin-adjusted calcium and 1.45 mmol/L in ionized calcium. For pregnant women with PHPT and calcium above these cut-off concentrations, parathyroidectomy, preferentially in the second trimester, is recommended. Medical treatment for hypercalcaemic PHPT is limited and requires individual decision making. Immediately after delivery, hypercalcemia may worsen in women with PHPT.

**Summary:**

Clinical care of women with HypoPT and PHPT in pregnancy and lactation requires intensive surveillance and consideration of the specific changes in bone and mineral metabolism during these times.

## Introduction

Changes in bone and mineral metabolism during pregnancy and lactation require consideration in the clinical care of women with parathyroid disorders [1–3]. Recent developments, including the availability of long-acting parathyroid hormone (PTH) replacement therapy, revised European Society of Endocrinology (ESE) hypoparathyroidism (HypoPT) guidelines, and accumulating evidence regarding primary hyperparathyroidism (PHPT) management during pregnancy, warrant updated practical information for clinicians.

 [4–9]. In this narrative review, we summarize and discuss current evidence regarding HypoPT and PHPT in pregnancy and lactation. Based on original data, expert statements and guidelines in this field, we provide guidance for patient care.

### Changes in Bone and Mineral Metabolism in Pregnancy and Lactation

Skeletal and related hormonal changes throughout pregnancy and lactation have been extensively reviewed elsewhere [1–3]. In brief, during pregnancy intestinal calcium absorption doubles to meet the calcium demands of the fetus while little if any skeletal resorption of maternal bones occurs. During lactation, maternal bone resorption is increased to provide sufficient minerals for breast milk resulting in a significant decrease of maternal bone mineral density (BMD), particularly at trabecular bone sites with e.g., a loss of lumbar BMD after 6 months of roughly 10% [2]. Maternal BMD reaches pre-pregnancy or early postpartum values 12 months after weaning, so that parity and lactation have no adverse long-term effect on maternal bone health. The above described changes in bone and mineral metabolism are mediated by hormonal changes in pregnancy and lactation. These include, amongst others, a two to five-fold increase in calcitriol (i.e., 1-25-dihydroxyvitamin D, the so-called active vitamin D hormone) during pregnancy with normalization during lactation, a decrease in PTH to low normal levels or slightly below the reference range in pregnancy and lactation, and a significant increase in PTH related protein (PTHrP) in pregnancy with a further increase during lactation. Increased estradiol concentrations in pregnancy rapidly and significantly decline during lactation, while all these hormonal changes normalize gradually after weaning.

Total calcium significantly decreases in pregnancy due to low albumin concentrations and hemodilution [1]. Therefore, ionized (free) calcium and/or albumin-adjusted calcium should be determined in pregnancy for assessment of calcium status. Whenever available and analytically reliable, ionized (free) calcium concentrations should be the preferred parameter, in particular in unclear, critical or challenging cases, as there are certain limitations of formulas to calculate albumin-adjusted calcium concentrations, e.g., limitations of albumin assays, the incorrect assumption of a completely linear relationship between albumin and total calcium, and ignoring other determinants of albumin binding (e.g. pH) [10–14]. After delivery, total calcium concentrations normalize and can be used for clinical decision making during lactation [1].

### Chronic Hypoparathyroidism

HypoPT is characterized by hypocalcemia with reduced or inappropriately low PTH concentrations. This orphan disease (prevalence of 6.4 to 38/100.000) is mainly (at least 75–80%) a consequence of anterior neck (thyroid) surgery, but can also be due to various non-surgical causes including, amongst others, various genetic diseases, autoimmunity, or unclarified (idiopathic) pathologies [4]. Conventional treatment is primarily based on activated vitamin D analogues and sufficient calcium intakes, but PTH replacement therapy is nowadays an attractive therapeutic option with promising long-term outcomes for renal function, quality of life with considerably reduced pill burden, and excellent biochemical disease control [4, 15].

### Chronic Hypoparathyroidism and Pregnancy Outcomes

The diagnosis of HypoPT is usually established before conception. Certain diseases and syndromes that cause or are associated with HypoPT such as autoimmune polyendocrinopathy candidiasis ectodermal dystrophy (APECED), may affect fertility [16–18]. Published evidence on use of assisted reproductive treatment in HypoPT is very limited, but only a minority of pregnant women with HypoPT required such interventions (e.g., 12% in one cohort study) [18, 19]. Overall, there is no solid evidence that HypoPT per se significantly affects fertility, and registry data document no difference in birth rates in women with HypoPT as compared to age-matched controls [20].

Table 1 shows several registry studies and case series (restricted to reports with at least 10 pregnancies) on maternal and fetal pregnancy outcomes in women with HypoPT [16, 19–27]. Collectively, most pregnancies of women with HypoPT were uncomplicated and resulted in the delivery of a healthy baby [28]. Findings from the two largest studies with detailed data on pregnancy outcomes show that there were significant risk increases for women with HypoPT as compared to controls for induction of labor and lower birth weight in the study by Björnsdottir et al., and for preterm births, blood transfusions and congenital anomalies in the study by Hochberg et al. [20, 22]. Regarding clinical significance, blood transfusions and congenital anomalies occurred in less than 5% of the cases in the study by Hochberg et al., while there was no difference in congenital malformations in the study by Björnsdottir et al. [20, 22]. For all other maternal, fetal and neonatal outcomes (e.g., mortality or pre-eclampsia), there was no risk increase in HypoPT [20, 22]. Notably, neither of these two large registry studies found a significant difference in the mode of delivery [20, 22].

Overall, although women with chronic HypoPT appear to have slightly increased risks for selected maternal and neonatal outcomes, the available evidence consistently suggests that the vast majority experiences uncomplicated pregnancies when calcium homeostasis is carefully maintained.

**Table 1:** Case series and registry studies on pregnancy outcomes in chronic hypoparathyroidism (restricted to reports with at least 10 pregnancies)

First author (year)	Number of women with HypoPT	Number of pregnancies in women with HypoPT	Main findings regarding pregnancy outcomes
Ali (2025)	9	18	Live birth rate was 83.3%. Greater risk for preterm delivery and neonatal hypocalcemia in nonsurgical versus postsurgical HypoPT
Hochberg (2023)	Not indicated	204	As compared to non HypoPT pregnancies, pregnancies in women with HypoPT were at significantly increased risk of preterm births (19.1% vs. 7.2%; OR: 2.49, 95% CI: 1.74–3.54), blood transfusions (4.9% vs. 1.0%; OR: 4.07, 95% CI: 2.15–7.73) and congenital anomalies (4.4% vs. 0.4%; OR: 6.50, 95% CI: 3.31–12.75). No significant differences for various other pregnancy outcomes but no assessment of potential early miscarriages.
Laakso (2022)	Not indicated but 43 patients with APECD had 83 pregnancies	Not indicated but 60 pregnancies resulted in deliveries (with 2 stillbirths) and 78% of women with livebirths had HypoPT	Miscarriages were not associated with HypoPT.
Konca Degertekin (2022)	111	140	Of the 140 pregnancies, 98 healthy infants were delivered, 36 abortions occurred, and six pregnancies were ongoing at the time of data collection.
Björnsdottir (2021)	97	139 deliveries	HypoPT as compared to age matched controls was associated with increased risk of induction of labor (OR: 1.82 (95% CI) 1.13–2.94) and lower birth weight (β-coefficient − 188 g (95% CI) -312.2 to -63.8 g). No significant difference was found for multiple other pregnancy outcomes and overall birth rates.
Wang (2021)	19	25	Preterm delivery and miscarriage occurred in 17.4% and 13.0% of the pregnancies, respectively.
Marcucci (2021)	28 (25 with HypoPT and 3 with pseudo-HypoPT)	28	Maternal complications occurred in 32% of women with HypoPT. 12% of women with HypoPT underwent assisted reproductive treatment.
Hartogsohn (2020)	12	19	There were 17 live births and two abortions.
Hatswell (2015)	6	10	Intrauterine growth restriction was suspected in two pregnancies, preterm births occurred in two pregnancies, oligohydramnios complicated two pregnancies and 3 newborns were small for gestational age.
Callies (1998)	11	12	There were 11 live births and one stillbirth.

*OR* odds ratio, *95% CI* 95% confidence interval, *APECD* autoimmune polyendocrinopathy-candidiasis-ectodermal dystrophy, *HypoPT* chronic hypoparathyroidism

### Treatment of Chronic Hypoparathyroidism in Pregnancy and Lactation

Conventional therapy of HypoPT with activated vitamin D (calcitriol or alfacalcidol) and calcium is recommended for women with HypoPT during pregnancy and lactation with the aim to keep calcium levels at the lower end or lower half of the normal range [4, 5, 7, 9]. This treatment should thus remain initially unchanged when a woman with well-controlled HypoPT becomes pregnant [4]. Regarding clinical care of women with HypoPT in pregnancy and lactation, some studies reported on the respective disease course and treatment requirements [19, 21, 26, 27]. In general, unpredictable dose adjustments of conventional therapy as well as changes in calcium levels are frequently reported in pregnancy in either direction with no consistent pattern or clear trend throughout pregnancy [19, 21, 26, 27]. For example, one study reported that although average doses did not change during pregnancy, there was a 20% increase or decrease in activated vitamin D treatment doses in more than half of the pregnancies [27]. Furthermore, serum phosphorus, magnesium, and 25-hydroxyvitamin D concentrations should be kept in the normal reference range as generally recommended for regular treatment of HypoPT [4]. As PTH-mediated renal magnesium absorption is reduced in HypoPT and magnesium requirements further rise during pregnancy, particular attention should be given to magnesium [1, 29]. To our knowledge, there are no clinical studies published that specifically investigate potential changes in calcium requirements during delivery. Nevertheless, in cases of prolonged labor or clinical signs of maternal muscle exhaustion, we believe that it may be reasonable to measure ionized calcium concentrations. Depending on ionized calcium concentrations, HypoPT treatment should be adjusted, if indicated, including consideration of intravenous calcium gluconate for clinically severe hypocalcemic women.

Following delivery, there is an acute shift in calcium homeostasis arising from the removal of the placenta as a major endocrine organ and the cessation of fetal calcium transfer. This is reflected by a significant rise in serum calcium from pregnancy to postpartum in most studies of women with HypoPT. Hartogsohn et al. reported an increase in ionized calcium from 1.20 to 1.32 mmol/L, while Wang et al. observed an increase in total serum calcium from 1.99 ± 0.11 to 2.57 ± 0.34 mmol/L resulting in hypercalcemia in 41.7% of patients after delivery [21, 26, 27]. Therefore, significant dose reductions in conventional therapy of HypoPT are usually required after delivery. The majority of women with HypoPT choose to breastfeed, and breastfeeding should not be discouraged solely because of the presence of HypoPT [26, 27]. In rare cases, breastfeeding women with pre-existing HypoPT before pregnancy may even be free of any requirement for conventional therapy [1, 26]. At the end of lactation and after weaning, conventional therapy needs to be gradually increased again in most women, usually to the dosage requirements before pregnancy [1, 4, 27]. Due to these changes in treatment needs and disease control of HypoPT throughout pregnancy, it is recommended by expert groups and guidelines to pursue regular active surveillance of HypoPT patients throughout pregnancy and lactation [4, 5, 7, 9]. Clinical visits with laboratory measurements (including at least calcium and creatinine/GFR measurements) should be regularly performed throughout pregnancy and lactation (e.g., every 3 to 4 weeks), and even more frequently immediately before and after delivery (e.g., weekly in the 4 weeks before and after delivery) [4]. After weaning, dosage requirements for treatment of HypoPT usually increase, but the time course of this change in treatment needs may vary considerably [30]. PTHrP production may promptly cease around the time of weaning with an immediate increase in dosage requirements [30]. By contrast, there is also a case report of a woman who thought that her HypoPT had been cured by lactation, as she needed no treatment for about one year after weaning, until she suddenly developed hypocalcemic symptoms and required pre-pregnancy doses of calcium and calcitriol [30]. Another woman appeared to be permanently cured from HypoPT after weaning due to persistent production of PTHrP by her breasts [30]. In view of these variable disease courses and being aware that there is no clear recommendation for surveillance of HypoPT after weaning in current guidelines, we personally consider it reasonable to perform a clinical visit and laboratory measurements for HypoPT within the first 1–2 weeks after weaning and thereafter every 4 to 8 weeks for the first year after weaning [4, 8, 30]. Interestingly, one study reported that clinical symptoms of HypoPT, i.e., cramps/tetany and paresthesia, were less common in pregnancy and lactation as compared to pre-pregnancy [19].

PTH replacement therapy with the currently only approved drug palopegteriparatide for the treatment of non-pregnant patients with HypoPT, may become increasingly used in the near future, raising questions regarding its safety and potential use during pregnancy and lactation [4, 15]. According to existing knowledge, PTH may not cross the placenta and may thus not directly affect the fetus [1]. The European Medicines Agency (EMA) states that due to limited data a decision to initiate or discontinue treatment with palopegteriparatide in pregnancy should take possible risks versus benefits into account [1, 4]. As palopegteriparatide is not orally absorbed, it is unlikely to adversely affect the breast-fed child. Four case reports have already documented the off-label use of PTH replacement therapy during pregnancy in HypoPT patients resulting in healthy babies and no significant pregnancy complications [6, 31–33]. The authors of this article are aware of a few additional unpublished cases of pregnant women with use of PTH replacement therapy and favourable outcomes. Given the limited evidence, publication of all available cases and participation in prospective studies, such as the “Global Pregnancy Registry to Assess Maternal, Fetal, and Infant Outcomes Following Exposure to YORVIPATH^®^ (Palopegteriparatide) During Pregnancy and Breastfeeding” (Clinicaltrials.gov ID: NCT07345494), are strongly encouraged. At present, we cannot generally recommend PTH replacement therapy in pregnancy and lactation, but it may be considered for individual patients based on risk benefit considerations [4]. If PTH replacement therapy is started pre-pregnancy due to inadequate disease control, stopping PTH replacement therapy and re-starting previously insufficiently effective conventional therapy in or shortly before pregnancy may also pose a risk for the mother and the fetus, and this must be weighed against the limited safety and effectiveness data of PTH replacement therapy in pregnancy.

Thiazides should be discontinued before pregnancy and though they could be considered for cautious use in the second and third trimester, this may hardly be justified and useful [4, 34]. Native vitamin D supplementation with cholecalciferol (vitamin D_3_) or ergocalciferol (vitamin D_2_) with e.g., 800 to 2000 international units (IU) per day should be given in addition to any treatment for HypoPT (also in addition to activated vitamin D) to ensure a sufficient vitamin D status [4]. This is also in line with the suggestion of the Endocrine Society for general empiric vitamin D supplementation in all pregnant women [4, 35, 36].

Interdisciplinary care of women with HypoPT during preconception, pregnancy and lactation is critical, including active regular surveillance due to unpredictable changes in disease control and treatment requirements. Newborns of mothers with HypoPT may be at increased risk of hyper- and hypocalcemia. Thus, it is recommended to measure ionized calcium in the newborns every second day for the first week of life, but if calcium was well controlled during pregnancy, fewer measurements may be justified [5–7]. General recommendations for HypoPT during preconception, pregnancy and lactation are shown in Table 2.

**Table 2:** Recommendations for HypoPT during preconception, pregnancy and lactation

Preconception	Ensure good disease control; inform on potential pregnancy risks in HypoPT but also mention that most women with HypoPT have uncomplicated pregnancies and give birth to healthy babies.
	Counselling regarding frequent surveillance during pregnancy and lactation and potential changes in treatment requirements; discontinue thiazides before pregnancy; PTH replacement therapy with palopegteriparatide may be continued during pregnancy based on individual risk-benefit considerations.
Pregnancy	Regular surveillance including laboratory measurements every 3 to 4 weeks and weekly in the month before delivery.
	Continue conventional therapy with activated vitamin D and calcium.
	Target a calcium level at the lower end of the normal range.
Lactation	Anticipate reduced dosage requirements after delivery; breastfeeding should not be discouraged.
	Regular surveillance including laboratory measurements every week in the first month after delivery and then every 3 to 4 weeks during breastfeeding.
Neonatal care	Measure ionized calcium in the newborns every second day for the first 1 week of life, but intensity of this calcium monitoring should depend on maternal calcium control during pregnancy.
	Pursue routine vitamin D supplementation in the newborn according to national guidelines

### Primary Hyperparathyroidism

PHPT is characterized by an elevated serum calcium in the presence of elevated or inappropriately normal PTH [37]. PHPT is mainly caused by clonally dysregulated overgrowth of one or more parathyroid glands with reduced expression of the calcium sensing receptor (CaSR) [38]. Most cases of PHPT occur sporadic in postmenopausal women, but about 10% are due to genetic diseases, e.g., multiple endocrine neoplasia syndromes, hyperparathyroidism jaw-tumor syndrome, or familial isolated PHPT [38]. Therefore, genetic testing should be considered in young patients with PHPT (e.g., below 30 years), and in those with multiglandular disease, family history of hypercalcemia and/or syndromic diseases [37, 38]. PHPT can be cured by surgical removal of the enlarged parathyroid gland(s), which is generally recommended for symptomatic patients and for asymptomatic patients with marked hypercalcemia, skeletal or renal disease involvement and/-or age below 50 years [37]. Considering this age cut-off for surgery and potential risks of PHPT in pregnancy, it is recommended that pregnancy should be deferred until PHPT has been cured by parathyroidectomy and calcium levels are normalized [5, 7]. PHPT may not significantly affect fertility, which is supported by a large retrospective cohort study that showed no significant difference in pregnancy rates in women with PHPT versus age-matched controls [39]. The incidence of PHPT in pregnancy is not clearly established. PHPT may roughly occur in around 1 in 1,000 pregnancies but data are very inconsistent with one registry study from India reporting a prevalence of 2.1% of gestational PHPT [39–42].

### Primary Hyperparathyroidism and Pregnancy Outcomes

Potential clinical symptoms of PHPT in pregnancy are unspecific and may resemble common complaints of pregnant women such as nausea, vomiting, polyuria, dehydration or fatigue [5, 43]. Fetal osmotic polyuria may lead to polyhydramnios and marked maternal hypercalcemia with increased risk of nephrolithiasis, acute kidney injury, pre-eclampsia and pancreatitis [5, 43, 44]. As fetal parathyroid glands may be suppressed due to hypercalcemia of the mother, this may cause neonatal hypocalcemia with seizures after birth [45, 46].

Large registry studies on PHPT in pregnancy suggest that women with mild hypercalcemia, e.g., around 2.6 to 2.8 mmol/L in albumin adjusted or total calcium, are not at significantly increased risk of maternal or fetal pregnancy complications [39, 40, 47]. Earlier and mostly smaller studies reported on significantly increased risk of pregnancy losses and certain maternal and fetal pregnancy complications in PHPT, which may be attributed to (publication) bias and/or reporting or detection of particularly severe cases [43, 46, 48–50]. Some, but not all, studies reported on a dose-response relationship between serum calcium levels and adverse pregnancy outcomes, with no risk increase at mild hypercalcemia but increased risk with higher calcium levels [46, 48]. In the absence of a clearly defined threshold for this risk increase, several studies and expert groups suggest using a cut-off concentration of approximately 2.85 mmol/L in albumin-adjusted calcium and 1.45 mmol/L in ionized calcium [5, 7]. This is roughly in line with an increase in albumin-adjusted.

serum calcium of 0.25 mmol/L above the upper limit of normal, which is the threshold for recommending parathyroidectomy in non-pregnant PHPT patients [37].

### Indications for Surgical Treatment of Primary Hyperparathyroidism in Pregnancy

In view of the general recommendation for parathyroidectomy in PHPT patients below the age of 50 years, the question is not whether but when the optimal timing of parathyroid surgery in pregnant and lactating women suffering from PHPT is [37]. Systematic reviews of observational studies conclude that neonatal outcomes may be significantly improved when comparing PHPT patients with surgical versus conservative treatment in pregnancy [46, 51–53]. For example, in a systematic review including 382 cases of PHPT with 108 parathyroidectomies in pregnancy, infant complication rates (i.e., death, requiring neonatal intensive care unit stays, calcium supplementation or intravenous hydration, tetany, growth retardation or respiratory distress) were significantly lower in women who underwent surgery versus conservative treatment (9.1% versus 38.9%) [51]. Regarding timing of parathyroidectomy in pregnancy, the lowest neonatal complication rate was observed for surgeries in the second trimester (e.g., 4.5% for surgery in the second versus 21.1% in the third trimester) [51]. In general, surgery in pregnancy, if indicated, is often scheduled for the second trimester to avoid risks of teratogenicity and abortions in the first trimester and induction of labor with preterm deliveries in the third trimester. Regardless of the stage of pregnancy, the neonatal outcome was always better when comparing surgery with conventional treatment in PHPT [51]. Significantly reduced risk of neonatal complications in pregnant women with PHPT who were treated surgically versus conservatively were also reported by another meta-analysis in 163 and 185 PHPT patients, in the surgical and non-surgical group, respectively [46]. That meta-analysis did not document significant group differences in some maternal outcomes, such as nephrolithiasis or pancreatitis, but reported that certain obstetric complications were significantly reduced in the surgical group: preterm labor (9.2% versus 21.6%), preeclampsia/eclampsia (3.7% versus 10.3%) and pregnancy loss (1.3% versus 15.3%) [46]. Interestingly, hyperemesis gravidarum occurred more frequently in the surgical group (11.0% versus 2.7%) [46]. In view of the above mentioned evidence on the effectiveness of parathyroidectomy in pregnancy and the dose response curve of serum calcium and adverse pregnancy outcomes, surgery, preferably in the second trimester, is recommended for pregnant women with marked hypercalcemia, e.g., albumin-adjusted calcium > 2.85 mmol/L and/or > 0.25 mmol/L above the upper limit of normal and/or ionized calcium > 1.45 mmol/L [5, 7, 37]. Decision on therapy should be made in an interdisciplinary board of gynecologists, endocrinologists, surgeons, anesthetists and if necessary pediatricians and radiologists.

### Preoperative Imaging and Parathyroidectomy in Pregnancy

Preoperative localization of pathologic parathyroid glands is critical for the success of parathyroidectomy and concordance of two different imaging methods would be optimal [5]. Given the potential adverse effects of several imaging modalities during pregnancy, including radiation exposure and the use of contrast agents that may cross the placenta, the international PHPT guidelines recommend restricting preoperative imaging to ultrasonography [37]. Consistent with these recommendations, most reported cases of preoperative imaging during pregnancy relied exclusively on ultrasonography without additional or alternative imaging methods [51]. Other experts argue that further imaging methods, i.e. ^99m^Tc-methoxyisobutylisonitrile (^99m^Tc-MIBI) scans and MRI, but in specific selected cases also sestamibi single-photon emission CT (SPECT-CT), 18 F-Fluorocholine PET/CT or methionine PET-CT/ (with modifications to protocols, i.e., low-dose protocols, to minimize fetal radiation exposure) could be considered after careful considerations of potential risks and benefits [5, 54, 55]. For example, in a case report, 18 F-Fluorocholine PET/CT with an ultra-low dose protocol was performed in a pregnant woman with PHPT at 16 weeks´ gestation to localize an intrathyroidal cystic parathyroid adenoma [56]. ^99m^Tc-MIBI has already been used in several published cases of PHPT in pregnancy as the radiation dose is below the threshold associated with fetal harm, in particular if additional precautions (e.g. low dose, urinary catheter for the mother, etc.) are undertaken [6, 57–59]. Ultrasonography-guided parathyroid fine needle aspiration (FNA) cytology and PTH wash-out measurements may also be useful for localization studies and have been successfully performed in pregnancy [60].

Parathyroidectomy in pregnancy should ideally be performed as a minimally invasive procedure with the use of intraoperative PTH monitoring with a > 50% drop 10 min after excision indicating surgical success [5, 6]. Bilateral neck exploration may be required in hereditary forms of PHPT with multiglandular disease [5, 7, 37]. Minimally invasive image-guided thermal ablation techniques such as radiofrequency ablation (RFA) and microwave ablation (MWA), or ethanol ablation have also been successfully used in selected cases [60–63].

### Conservative Treatment of PHPT in Pregnancy

Conservative treatment of hypercalcemia due to PHPT includes sufficient oral (and/or intravenous) hydration and avoidance of overwhelming oral intake of calcium by diet and/or supplements [6]. Medical treatment of hypercalcemia is a potential bridging therapy until surgery and should only be considered after carefully weighing the risks and benefits. Calcitonin injections can be used during pregnancy as calcitonin does not cross the placenta and can rapidly lower calcium by direct inhibition of osteoclasts, but it is often only effective for a few days due to tachyphylaxis [1, 5, 6, 30, 44]. Oral phosphate has also been used in pregnancy but with limited effectiveness and risks of diarrhea, hypokalemia and soft tissue calcifications [1, 30]. Cinacalcet, an oral CaSR agonist, has already been used in several pregnant women and some experts suggest that its use may be considered in pregnant women with marked hypercalcemia (e.g., albumin-adjusted calcium concentrations > 3.0 mmol/L) [6, 64, 65]. Though cinacalcet crosses the placenta and it might hypothetically interfere with CaSRs of the placenta that regulate fetal-placental calcium transfer, its use was not associated with any teratogenic effects so far [65]. It may, however, cause dose-limiting gastrointestinal adverse effects, particularly nausea and vomiting, that may reduce tolerability and limit its effectiveness to normalize serum calcium [65]. Moreover, some newborns of mothers using cinacalcet in pregnancy suffered from hypocalcemia (i.e., 18.8% in a literature review) [65]. Bisphosphonates are formally contraindicated in pregnancy as they also cross the placenta and may thus potentially adversely affect fetal endochondral bone development [1, 30]. A systematic review of 108 pregnancies with use of bisphosphonates for various reasons, though, concluded that evidence remains inconclusive for a direct causal relationship between exposure to bisphosphonates before and during pregnancy and fetal alterations [66]. Therefore, some experts (including us) would consider the use of bisphosphonates in pregnant women with otherwise uncontrollable severe hypercalcemia (e.g., albumin-adjusted calcium concentrations > 3.5 mmol/L) [6, 7]. Denosumab does also cross the placenta and as it caused adverse effects such as an osteopetrotic-like phenotype in animals, it should not be used during pregnancy (or only as the very last option), though it has been used for osteoporosis treatment in one woman during early pregnancy with no apparent adverse effect on the infant [30, 67].

Surveillance of PHPT patients with clinical visits and laboratory measurements (including at least calcium and creatinine/GFR measurements) is recommended according to expert statements every 4 weeks during pregnancy and every 4 and 8 weeks during lactation starting in the first week after delivery [5–7].

Newborns of mothers with hypercalcemic PHPT during pregnancy may be at increased risk of hypocalcemia that usually occurs on day 2 or 3 postpartum (this risk may be related to the degree of maternal hypercalcemia during pregnancy). It is recommended to measure ionized calcium in the newborns every second day for the first 1 to 2 weeks of life, but if calcium was well controlled during pregnancy, fewer measurements may be justified [5–7]. Active vitamin D treatment should be considered in case of hypocalcemia and standard neonatal vitamin D supplementation according to national guidelines should be pursued in all newborns [5–7].

### Treatment of PHPT After Delivery/During Lactation

In women with prevailing PHPT after delivery, it should be considered and anticipated that hypercalcemia may worsen immediately after delivery at the beginning of lactation, mainly driven by increased PTHrP production during lactation together with cessation of placental calcium transfer [30]. It is recommended to perform parathyroid surgery soon (i.e., in the first few weeks) after delivery when the mother has fully recovered and before any new pregnancy [5, 37]. Regarding medical treatment of hypercalcemia during lactation, it should be taken into account that cinacalcet is transferred to breast milk with a high milk to plasma ratio warranting a careful risk/benefit consideration to either discontinue breastfeeding or cinacalcet [5, 64]. Recommendations for women with PHPT during preconception, pregnancy and lactation are shown in Table 3.

**Table 3:** Recommendations for PHPT during preconception, pregnancy and lactation

Preconception	Pregnancy should be deferred until surgical cure of PHPT.
Pregnancy	Regular surveillance including laboratory measurements every 4 weeks.
	Consider parathyroidectomy, preferably in the second trimester, for patients with marked hypercalcemia, e.g., albumin-adjusted calcium > 2.85 mmol/L and/or > 0.25 mmol/L above the upper limit of normal and/or ionized calcium > 1.45 mmol/L.
	Ensure sufficient hydration and avoidance of overwhelming oral calcium intake.
	Consider medications to lower calcium in marked hypercalcemia; e.g., cinacalcet if albumin-adjusted calcium is > 3.0 mmol/L or bisphosphonates if albumin-adjusted calcium is > 3.5 mmol/L and cannot be sufficiently treated by other measures. Calcitonin may also be used to lower calcium but is usually only effective for a few days.
Lactation	Regular surveillance including laboratory measurements every 4 to 8 weeks.
	Surgery for PHPT should be pursued a few weeks after delivery.
Neonatal care	Measure ionized calcium every second day in the first 1 to 2 weeks, but intensity of this calcium monitoring should depend on maternal calcium control during pregnancy.
	Consider activated vitamin D treatment if hypocalcemia is detected in the newborn.
	Pursue routine vitamin D supplementation in the newborn according to national guidelines.

## Conclusions

Parathyroid disorders in pregnancy and lactation require an understanding of the specific disease course and surveillance as well as treatment requirements during these times. Interdisciplinary care and careful patient management at specialized tertiary care centers are critical to ensure that pregnancy is uncomplicated and to optimize maternal and neonatal outcomes. Future prospective registries and international collaborative studies are needed to strengthen the evidence base and refine management recommendations for women with parathyroid disorders during pregnancy and lactation.

## Data Availability

No datasets were generated or analysed during the current study.
